# Identification and Stability Analysis of Mine Goafs in Mineral Engineering Based on Multi-Survey Data

**DOI:** 10.3390/s25092776

**Published:** 2025-04-28

**Authors:** Huihui Jia, Mengxi Zhang, Qiaoling Min, Shuai Han, Jingyi Zhang, Mingchao Li

**Affiliations:** 1 State Key Laboratory of Hydraulic Engineering Intelligent Construction and Operation, Tianjin University, Tianjin 300350, China; magic-jia@163.com (H.J.); zhangmx@tju.edu.cn (M.Z.); zzjjyy_1223@tju.edu.cn (J.Z.); lmc@tju.edu.cn (M.L.); 2 Geological Disaster Monitoring and Early Warning Technology Innovation Center of Hebei Province, Chengde 067000, China; 3 Department of Building and Real Estate, Hong Kong Polytechnic University, Hong Kong 999077, China; shuaihan@polyu.edu.hk

**Keywords:** mineral engineering, mine goaf, multi-survey data, stability analysis, geological disaster

## Abstract

**Highlights:**

- Integration of multi-survey data from remote sensing and 3D laser scanning to identify risky mine goafs.
- Construction of detailed 3D models of complex mine goafs using FLAC^3D^ for numerical simulation.
- Verification of numerical simulation results with on-site investigations to assess geological disasters.
- Effective technical means for detailed surveys and stability assessments of mineral engineering in complex mine goafs are provided.

**What are the main findings?**

- The integration of remote sensing and 3D laser scanning enables precise location and shape reconstruction of complex mine goafs.
- Numerical simulations reveal the stress distribution, failure mechanisms, and surface deformation responses of mine goafs, highlighting their impact on mine stability.
- On-site investigations confirm the geological hazards predicted by the numerical models, validating the reliability of the proposed methods.
- The research provides a comprehensive technical approach for assessing and mitigating risks associated with complex mine goafs.

**What are the implications of the main findings?**

- The proposed methods significantly improve our ability to identify and assess risks in mineral engineering with complex goafs, reducing the likelihood of geological disasters.
- The integration of remote sensing, 3D laser scanning, and numerical modeling offers a practical solution for detailed goaf surveys and stability evaluations.
- The findings can inform regulatory frameworks and safety standards for mining operations in regions with unregulated historical mining activities.
- The study advances the use of multi-survey data and numerical simulations in mining engineering, setting a precedent for future research and applications.

**Abstract:**

Unregulated underground group mining in China has led to problems such as unclear locations and complex shapes of mine goafs in mineral engineering, posing serious safety hazards for subsequent mining operations. This paper takes mineral engineering with complex mine goafs as the research object, integrates multi-survey data from surface deformation remote sensing monitoring and 3D laser scanning measurement to survey the area where the surface deformation rate reaches 14cm/ year, accurately identifies the location of risky mine goafs, and constructs detailed representations of the real shapes of the complex mine goafs inside the mineral engineering. The FLAC^3D^ 6.0 software is used to establish a 3D numerical simulation model of the mine goafs, fully considering the mining process, and conducting characteristic analysis of the stress distribution, failure range and surface deformation response of the mine goafs, revealing the impact of void deformation on the stability of the mine. The numerical simulation results are combined with on-site investigations to verify whether geological disasters have been caused by mine goafs. The research methods and results can provide effective technical means for the detailed survey and stability assessment of mineral engineering with complex mine goafs, which can help to reduce the risk of geological disasters in mines and improve the safety of mineral engineering.

## 1. Introduction

Mineral resources, as one of the key pillars of economic development, have garnered considerable attention. The continuous expansion of mineral extraction has led to a transition from surface open-cast mining to deep underground mining. As near-surface resources are gradually depleted, deep ore extraction faces numerous challenges, with safety issues being particularly urgent. The extensive mine goafs created by underground mining operations can induce various geological disasters, such as surface collapse, water inrush, ground subsidence, and falling roofs. These disasters pose serious threats to mine safety, resulting in significant casualties and property losses [1,2,3]. Therefore, the accurate acquisition of goaf spatial morphological characteristics is crucial for stability analysis. Goaf regions, shaped by factors including topography, geological settings, mining conditions, and overburden load types, exhibit diverse morphological configurations. Developing effective goaf surveying methodologies has thus become pivotal in addressing these challenges [4].

Current survey methodologies for goaf regions primarily include field engineering, geological investigations [5,6], geophysical exploration [7,8], and drilling [9,10], supplemented by deformation monitoring [11,12]. Internationally, nations such as the United States, Japan, and Russia predominantly utilize geophysical exploration techniques for goaf detection. In China, early-stage underground goaf surveys were primarily conducted using drilling methods; however, with technological advancements, geophysical exploration has become the predominant approach in recent years. This shift has led to the development of various high-precision techniques, including three-dimensional laser scanning, transient electromagnetic methods, microtremor detection, and advanced geological prediction systems [13,14,15]. The inherent challenges in goaf surveying arise from several factors: the unpredictable spatial distribution of voids, the structural complexity of geological formations, and the variable stress states in roof strata and surrounding rock masses. Due to these complexities, single-method approaches often prove inadequate for accurately characterizing goaf geological conditions. As a result, integrated survey strategies combining multiple techniques are increasingly being adopted in engineering practice to achieve an improved detection accuracy [16].

Stability assessments of goaf regions represent a fundamental aspect of mining safety management. Empirical evidence from field studies indicates that roof collapse in abandoned mine voids can induce significant surface subsidence and catastrophic ground failures, posing substantial risks to surface infrastructure [17]. In response to these challenges, significant research efforts have been devoted to developing advanced analytical approaches. He et al. [18] employed COMSOL Multiphysics to investigate goaf morphological evolution, establishing a predictive model for void development. Ke et al. [19] developed an innovative early warning system for goaf stability based on D-RES theory and an asymmetric fuzzy correlation cloud model, which integrates multiple geomechanical indicators through fuzzy interval quantification and analyzes their interactions using D number theory combined with RES theory. Further advancing the field, Li et al. [20] proposed a hybrid risk assessment methodology combining an improved arithmetic optimization algorithm (IAOA) with support vector machine (SVM) techniques, integrated with GoCAD-FLAC3D numerical simulations, demonstrating its superior performance through applications in multiple goaf projects. From an engineering perspective, deformation monitoring of abandoned mine workings serves as a critical component for safety early warning systems. Fan et al. [21] achieved precise goaf localization by integrating InSAR technology with the probability integral method (PIM), while Jia et al. [22] successfully characterized internal goaf structures using 3D resistivity tomography, providing essential data for comprehensive safety analyses. These methodological advancements collectively contribute to improved risk assessments and the management of abandoned mine workings.

Given the complex geometry of mine goaf regions, it is challenging for a single surveying technology to accurately identify these areas and construct detailed models of the mine goaf regions. To address this issue, this paper integrates multi-survey technology with an airborne–spaceborne–terrestrial integrated monitoring system, interprets the surface deformation of mining area based on InSAR time-series technology and unmanned aerial vehicle photogrammetry and three-dimensional laser scanning (LiDAR) technology, to identify and delineate the orientation of potential mine goaf regions, carry out micro-motion intelligent exploration and drilling on the surface to verify mine goaf regions, and use three-dimensional laser scanning technology to obtain a point cloud of cavity shape data for mine goaf regions during drilling, thus building a picture of the shape of mine goaf regions. Taking mineral engineering in Luanhe River, which includes complex mine goaf regions, as the research object, a case analysis is carried out, and a numerical model with mine goaf regions is constructed to analyze the stability of mine goaf regions. Combined with the numerical simulation results and a field investigation, the geological disasters caused by the existence of mine goaf regions are verified. The approach used in this research can provide an effective technical framework for carrying out comprehensive surveys and stability assessments of complex mine goaf regions, holding significant value for enhancing mineral engineering safety and mitigating geological disaster risks.

## 2. Data and Methods

### 2.1. Data

#### 2.1.1. Study Area and Geological Context

This study focuses on a mining project situated on the northern bank of the Luanhe River, covering an area of 2.0457 km^2^ with a mining depth ranging from 187 m to 585 m. The mining area exhibits a topographic gradient, sloping from north (highest elevation: 822.9 m) to south (lowest elevation: 516 m), following the regional geological strike. Geomorphologically, the site is classified as a low-mountain erosional structural landform, characterized by a northeast–southwest topographic trend aligned with fault structures. The central region comprises an intermontane valley—a densely populated zone—flanked by relatively undisturbed terrain to the east and west.

**Figure 1:**
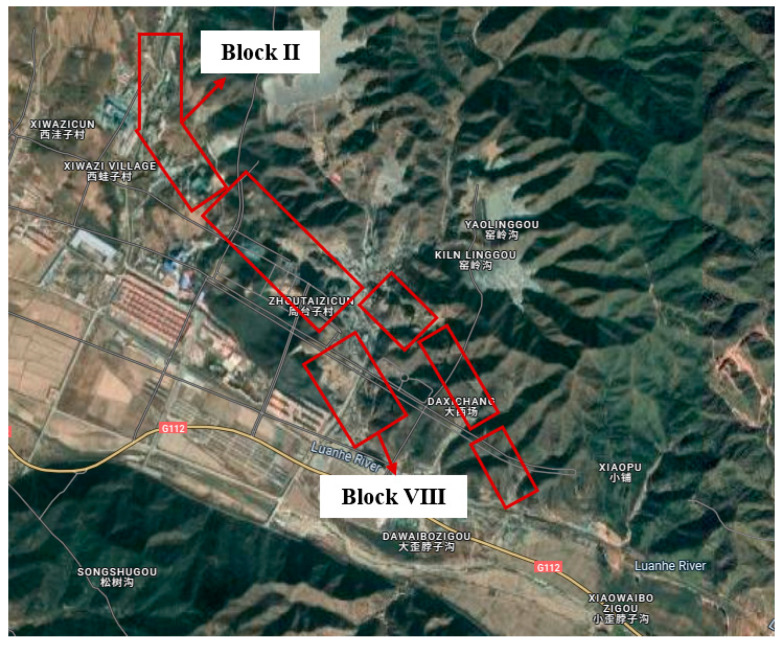
Partition distribution of each block’s area.

Historical mining operations have resulted in the formation of multiple goaf clusters with significant dimensional variability. Some cavities exhibit collapse features attributed to stress redistribution during subsequent excavations. Shallow, undocumented voids—legacies of early-stage unregulated mining—are also suspected to exist. To systematically evaluate these geotechnical challenges, including poorly constrained spatial distributions, heterogeneous void geometries, and vertical overlap configurations, the study area was partitioned into several geotechnical assessment zones (Figure 1) based on historical mining boundaries.

#### 2.1.2. Remote Sensing Data

The proposed airborne–spaceborne–terrestrial integrated monitoring system synergistically combines satellite remote sensing, unmanned aerial vehicle (UAV) photogrammetry, and light detection and ranging (LiDAR) technologies to capture high-resolution time-series point cloud data of surface subsidence. By employing advanced algorithmic inversion techniques and digital elevation model (DEM) superposition analysis, this methodology enables an accurate reconstruction of the spatiotemporal deformation patterns induced by underground goaf collapse. The processed datasets were subsequently integrated into three-dimensional goaf models, providing quantitative visualization capabilities for analyzing subsidence evolution dynamics. This multi-scale monitoring framework effectively bridges the gap between large-scale regional surveys and localized high-precision monitoring, significantly enhancing detection accuracy in mining areas.

(1) InSAR time-series data

**Figure 2:**
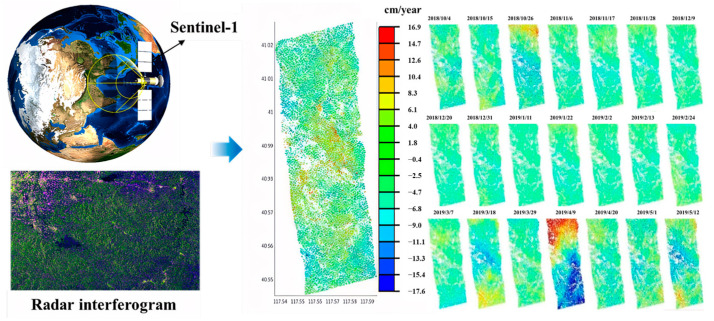
Annual average deformation rate in exploration area.

Large-scale deformation monitoring constitutes the foundational stage of goaf identification. We employed Persistent Scatterer Interferometric Synthetic Aperture Radar (PS-InSAR) technology to systematically map deformation anomalies throughout the study region. Our time-series analysis incorporated Sentinel-1A SAR imagery (11-day revisit cycle, 41.1° incidence angle), precisely co-registered with the 30 m resolution Shuttle Radar Topography Mission (SRTM) digital elevation model. The annual average subsidence rates were derived for the investigation zone characterized by a high-density goaf distribution and a close proximity to residential areas, peaking at 17.6 cm/year. Figure 2 presents the derived 11-day interval deformation velocity field, which clearly demonstrates substantial surface displacement patterns corresponding to underground mining operations. The analysis revealed progressive deformation accumulation, suggesting that exceeding critical thresholds could potentially trigger goaf roof collapse events.

(2) UAV imagery

Following the InSAR analysis, UAV-based photogrammetric surveys were conducted to obtain high-resolution digital terrain data covering potential goaf-affected surface areas. Temporal comparisons of multi-epoch digital terrain models enabled precise spatial delineation of suspected goaf zones. This approach provides centimeter-level orthorectified imagery that effectively compensates for InSAR decorrelation while enabling accurate deformation quantification via differential analysis of multi-temporal digital elevation models (DEMs) and digital surface models (DSMs).

**Figure 3:**
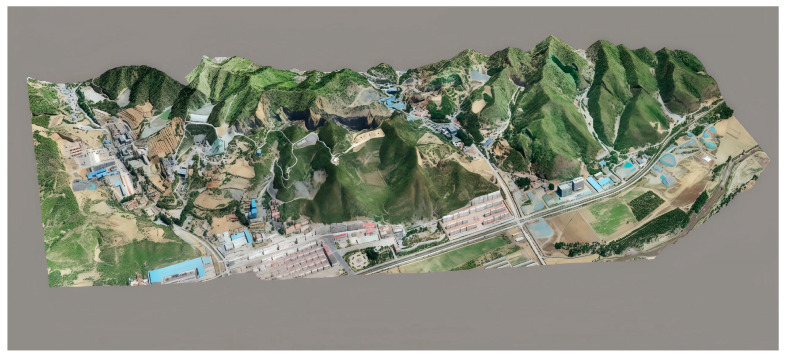
UAV photogrammetry map.

A UAV system integrated with a high-precision positioning module (GNSS) and onboard data logger was deployed for dense point cloud acquisition. Vegetation interference was eliminated through point cloud filtering, generating a bare-earth digital terrain model (Figure 3). The survey captured 1399 aerial images, producing 664,539,650 dense point cloud measurements with a ground control point error of 0.057 m. The resulting 3D model, covering 4.91 km^2^, comprises 132,067,457 polygons and 66,071,214 vertices.

**Figure 4:**
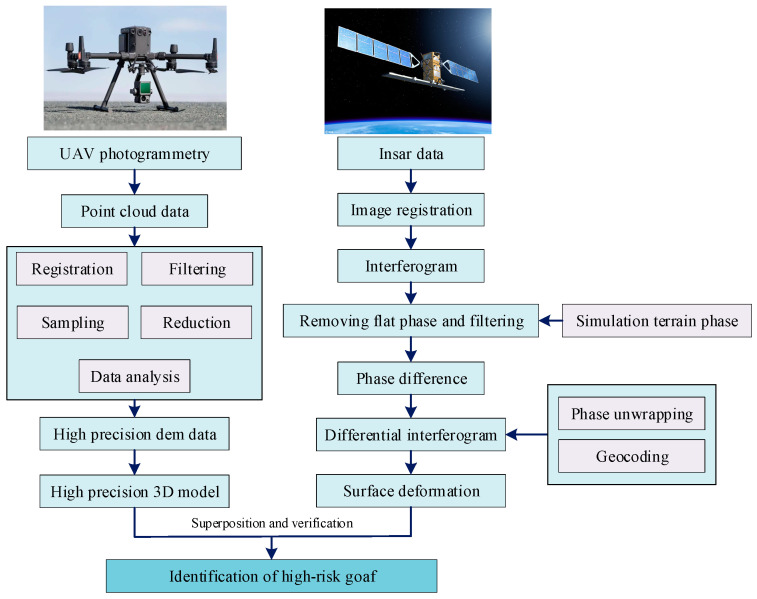
Data processing flow in InSAR and UAV methods.

High-risk mine goaf regions were identified and located using the InSAR and UAV methods, and the datasets should be aligned and overlapped according to the following process depicted in Figure 4. According to this process, the mine goaf regions were identified and the surface deformation rate was monitored. The warning level of the site was judged using the classification system in Table 1 below, and corresponding monitoring and counter measures are adopted.

(3) Terrestrial LiDAR scans

LiDAR 3D laser scanning technology was applied to acquire high-density point cloud data (1 cm accuracy) of potential goaf surface areas. Compared with the aforementioned surface deformation monitoring techniques (InSAR and UAV photogrammetry), this method achieves a higher precision in delineating goaf boundaries and provides a critical modeling foundation for subsequent stability analysis. Three-dimensional laser scanning technology is a long-distance fast ranging technology based on spatial lattice scanning technology and a laser non-reflective prism. The system employs a polygonal mirror rapid rotation scanning mechanism, generating completely linear, uniformly distributed, unidirectional, and fully parallel scanning laser point cloud lines. During operation, the laser pulse emitter transmits a beam of laser pulse signals that undergo diffuse reflection upon reaching the target surface. The return signal is captured by the receiver, enabling calculation of the time difference *t* between emission and reception. The distance *S* between the scanner and any surface point is then determined by:

(1) $$S=\frac{ct}{2}$$

where *S* is the distance from the scanner to the target point, *c* is the speed of light, and *t* is the time difference from when the laser receiver receives the pulse signal.

Three-dimensional laser scanning technology utilizes a precision clock-controlled encoder integrated within the scanning system to capture both the longitudinal and lateral scanning angles of laser pulses during measurement. This mechanism enables a precise determination of 3D coordinate values for each laser point on the target surface. By overcoming limitations inherent to conventional monitoring methods, including sparse monitoring point density, a limited measurement accuracy, prolonged monitoring cycles, susceptibility to sensor damage, and personnel safety risks in hazardous zones, this approach establishes a new paradigm for high-resolution geotechnical monitoring.

#### 2.1.3. Subsurface Scanning Data

The borehole-deployed Cavity-Aerial Laser Scanner (C-ALS) system, operating at a wavelength of 905 nm, demonstrates exceptional capabilities for underground void characterization. Key specifications include a 1 cm spatial resolution, 150 m maximum detection range (minimum: 0.5 m), a horizontal circle accuracy of 0.2° with a 0.1° angular resolution, and a 5 cm diameter probe housing a triaxial MEMS gyroscope (<1°/20 min azimuthal drift, 400°/s angular velocity range). This configuration enables 360° spherical scanning within a 150 m radius, which is particularly effective in inaccessible cavities, subsurface voids, and confined underground spaces. Integration of the instrument’s embedded 3D high-precision navigation sensors with existing geodetic control networks achieves comprehensive blind-zone-free 3D mapping. Post-acquisition processing through high-density point cloud software reconstructs the goaf morphology at the millimeter level, achieving a 1 cm spatial resolution in the final 3D models.

**Figure 5:**
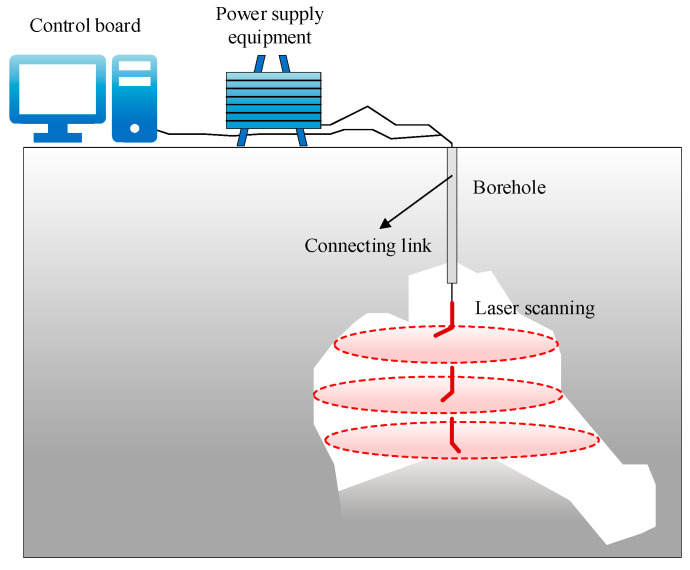
Schematic diagram of field test method.

The deployment of a 3D laser scanning probe within underground goaf regions enables precise delineation of their spatial morphology. The automated scanning system facilitates rapid and safe subsurface investigation by inserting the laser probe into pre-drilled boreholes. With a probe diameter of 50 mm, this configuration permits access to confined underground spaces and complex cavities through narrow exploration drillholes. The probe integrates a drilling camera module equipped with red LED illumination, facilitating real-time visual confirmation of borehole conditions and obstacle identification during descent. This visual guidance system proves particularly effective in locating cavity entrances obscured by collapsed debris or water infiltration. Upon entering the void, the scanning head automatically deploys, initiating 360° spherical scanning while simultaneously recording surface reflectance values. The field deployment configuration is illustrated in Figure 5. The acquired point cloud data undergo filtering through geometric reconstruction algorithms to generate millimeter-accuracy goaf models, providing critical model parameters for subsequent stability analysis.

### 2.2. Methods

#### 2.2.1. Integrated Surface Monitoring Techniques

**Figure 6:**
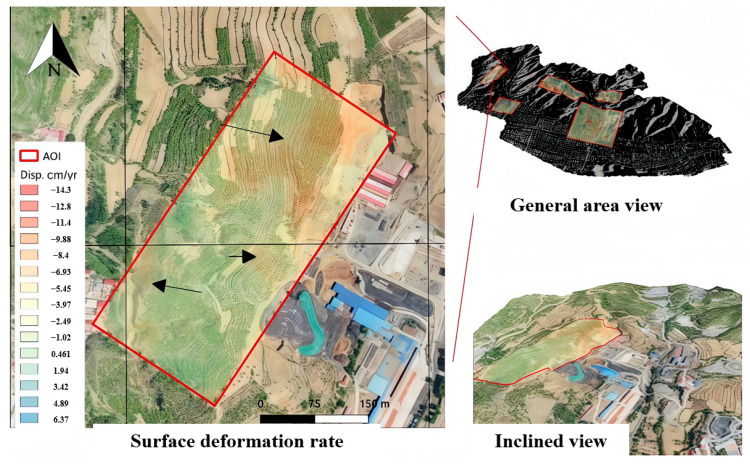
InSAR surface deformation.

The integrated processing of the three aforementioned datasets (InSAR, UAV photogrammetry, and LiDAR) compensates for individual technological limitations, enabling comprehensive analyses ranging from regional-scale reconnaissance to localized precision monitoring. The InSAR-derived deformation velocity field revealed four rapidly subsiding zones (peak rate: 14 cm/year), which were identified as high-risk goaf candidates (Figure 6).

**Figure 7:**
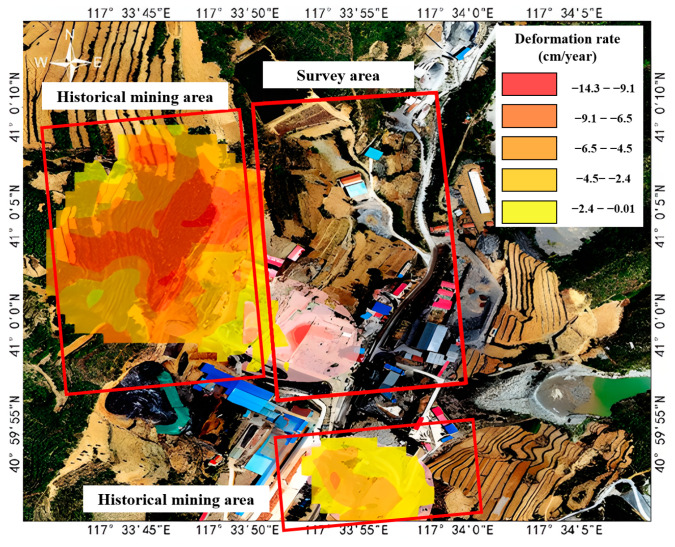
Identification of high-risk zones.

For the target investigation area, terrestrial LiDAR scanning was conducted across 11 stations, yielding 7.3 × 10^8^ raw point measurements. Post-processing workflows including point cloud registration, noise removal, vegetation filtering, and data decimation generated a triangulated irregular network (TIN), culminating in a 10 cm accuracy digital terrain model (DTM) raster. Cross-validation through differential analysis between LiDAR-derived DTM and UAV-generated DTM confirmed the consistency of the InSAR deformation patterns with photogrammetric observations. Multidisciplinary analysis of the synergistic monitoring results delineated high-risk zones (Figure 7), predominantly clustered in the southwestern sector of the mining area. Notably, elevated deformation rates exceeding 8 cm/year were also detected in extra-mine areas, particularly along hillslope terrain. It is worth noting that the deformation in the extra-mine areas is caused by the underground cavities formed in the early disorderly civilian mining stage.

#### 2.2.2. Subsurface Void Detection and Modeling

**Figure 8:**
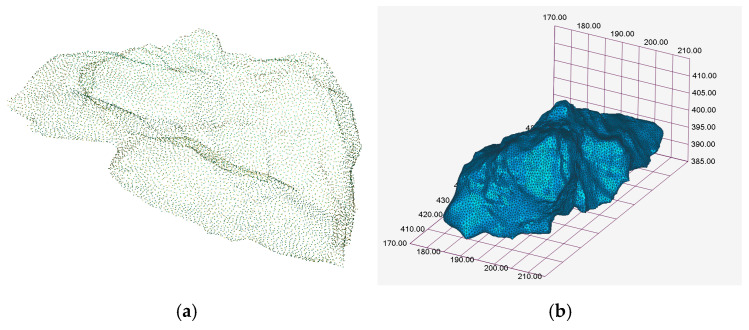
Accurate model of the mine goaf: (**a**) Point cloud data; (**b**) Refined spatial model.

Accurate reconstruction of goaf cavity models empowers mining engineers to conduct targeted stability assessments of underground void structures while precisely calculating backfill material requirements. The point cloud data acquired through 3D laser scanning enables the reconstruction of geometrically refined spatial models that faithfully represent subsurface cavities (Figure 8). These high-fidelity models capture critical geometric parameters including cavity morphology, roof overhang dimensions, and volumetric measurements. The reconstructed geometric models were subsequently imported into the FLAC^3D^ finite difference software for numerical model establishment.

#### 2.2.3. Numerical Modeling of Goaf Stability

Based on the precise goaf morphology obtained through the detection methodologies outlined in the previous section, a high-precision three-dimensional numerical model of the mine structure incorporating goaf geometries was constructed using FLAC^3D^ software. This model accounts for mining-induced stress evolution to analyze stress distribution patterns, failure zones, and surface deformation responses, thereby evaluating the destabilizing effects of goafs on overall mine stability.

The relatively flat terrain in the mining area exhibits surface elevations ranging from 447 m to 449 m. Borehole 3D laser scanning data from exploration boreholes ZK4, ZK5, and ZK6 in Block II, along with ZK10 in Block VIII, were utilized to establish goaf-specific numerical sub-models. The key spatial parameters of these goaf structures are detailed in Table 2.

The stability analysis of goaf regions employs the Mohr–Coulomb yield criterion, a widely adopted constitutive model in geotechnical engineering particularly suited for evaluating the global mechanical behavior of granular geomaterials under monotonic loading [23]. The mathematical formulation of the Mohr–Coulomb yield surface is expressed as:

(2) $$F=R_{mc}q-p\tan\varphi -c=0$$

where p is the equivalent compressive stress; q is the Mises equivalent stress; φ is the inclination angle of the Mohr–Coulomb yield surface on the stress surface, which is generally defined as the internal friction angle of the soil material; c is the cohesion of soil materials; and $R_{mc}$ controls the shape of the yield surface on the plane, which can be calculated according to the following formula:

(3) $$R_{mc}=\frac{1}{\sqrt{3}\cos\varphi }\sin(\theta +\frac{\pi }{3})+\frac{1}{3}\cos(\theta +\frac{\pi }{3})\tan\varphi$$

where θ denotes the generalized shear stress orientation angle, defined as $\cos(3\theta )=\frac{r^{3}}{q^{3}}$, where r represents the third deviatoric stress invariant $J_{3}$.

**Figure 9:**
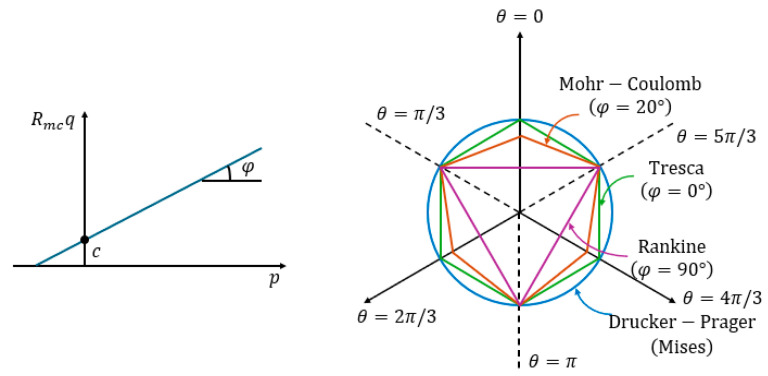
Mohr–Coulomb yield surface.

Figure 9 shows the shape of Mohr–Coulomb yield surface on meridian plane and plane, which can be compared with the Druker–Prager yield surface, Tresca yield surface, Rankine yield surface, and Mises yield surface.

The numerical modeling domain extends from the ground surface elevation of 449 m down to 340 m below the surface, with stratigraphic layers simplified as horizontal deposits. The model employs solid elements governed by the Mohr–Coulomb failure criterion, incorporating horizontal displacement constraints on the lateral boundaries, fixed vertical constraints at the base, and a stress-free ground surface. Rock mechanical parameters required for computation were obtained through laboratory tests conducted in accordance with the Regulation for Testing the Physical and Mechanical Properties of Rock, as listed in Table 3.

**Figure 10:**
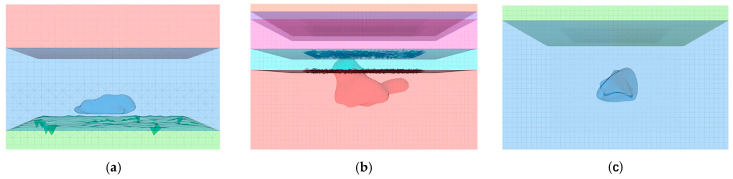
Numerical simulation model of mine goafs: (**a**) ZK4 in Block II; (**b**) ZK5 and ZK6 in Block II; (**c**) ZK10 in Block VIII.

For Block II, the goaf model in ZK4 (Figure 10a) spans depths from 449 m to 404 m, with dimensions of 40 m × 60 m × 45 m. This model comprises 45,800 elements (hexahedral and tetrahedral hybrid mesh) and 15,036 nodes. The goaf model in ZK5 and ZK6 (Figure 10b) cover depths from 447 m to 340 m, featuring dimensions of 150 m × 160 m × 107 m with 197,513 elements (hybrid mesh) and 91,331 nodes. In Block VIII, the goaf model in ZK10 (Figure 10c) extends from 447 m to 350 m depth, measuring 140 m × 160 m × 97 m with 160,280 elements (hybrid mesh) and 80,749 nodes.

## 3. Results

The investigation of stress distribution patterns across various mining levels facilitates a comprehensive analysis of ground pressure dynamics within the entire mining area. By examining the nonlinear deformation distribution and magnitude of surface subsidence, the destabilizing effects of goafs on regional stability are systematically evaluated.

### 3.1. Ground Pressure Distribution Characteristics

**Figure 11:**
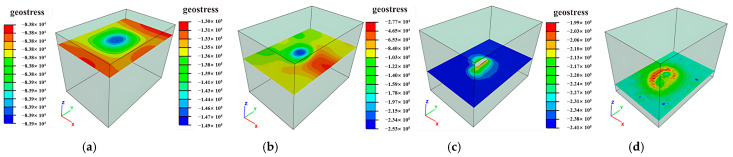
Distribution of ground pressure during mining of in Block II (ZK4): (**a**) hole position 440 m; (**b**) Hole position 430 m; (**c**) hole position 420 m; (**d**) hole position 410 m.

**Figure 12:**
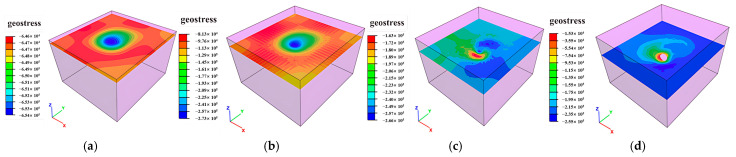
Distribution of ground pressure during mining of Block II (ZK5 and ZK6): (**a**) hole position 440 m; (**b**) hole position 430 m; (**c**) hole position 420 m; (**d**) hole position 410 m.

**Figure 13:**
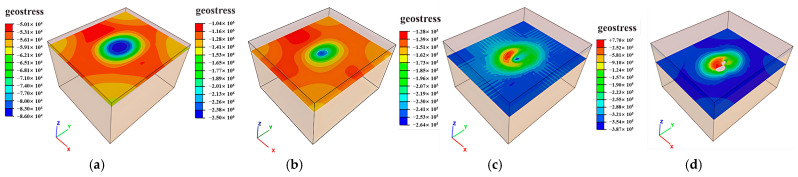
Distribution of ground pressure during mining of in Block VIII (ZK10): (**a**) hole position 440 m; (**b**) hole position 430 m; (**c**) hole position 420 m; (**d**) hole position 410 m.

Mining operations induce stress release and redistribution in surrounding rock masses. As shown in Figure 11, Figure 12 and Figure 13, the principal stress distributions in the ZK4, ZK5, ZK6 (Block II), and ZK10 (Block VIII) goaf models demonstrate progressive stress field evolution during excavation. Horizontal cross-sections were extracted at 10 m vertical intervals, revealing compressive stress concentrations predominantly at the geometric transitions between goaf sidewalls, with localized tensile stresses observed in the roof strata.

When microcracks develop within tensile stress zones, stress concentrations emerge at crack tips, significantly amplifying tensile stress concentration factors in roof strata. Given the inherently low tensile strength of fractured rock masses, such stress concentrations predispose roof rocks to progressive caving, with the extent of collapse directly correlating to tensile stress distributions. While global stability remains minimally affected by the limited spatial extent of maximum tensile stresses, blasting-induced vibration during mining operations accelerates crack propagation in roof strata. This mechanism leads to localized tensile failure and subsequent roof collapse, particularly in areas with pre-existing joint networks.

### 3.2. Deformation Distribution Characteristics

**Figure 14:**
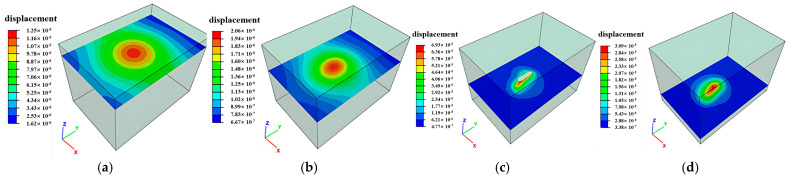
Deformation distribution of surrounding rock during mining of in Block II (ZK4): (**a**) hole position 440 m; (**b**) hole position 430 m; (**c**) hole position 420 m; (**d**) hole position 410 m.

**Figure 15:**
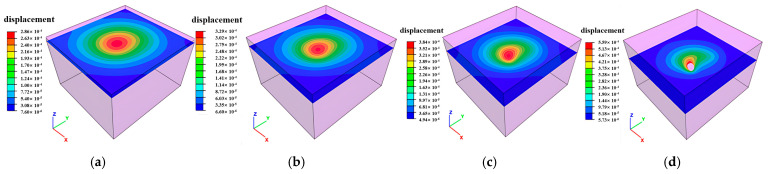
Deformation distribution of surrounding rock during mining of in Block II (ZK5 and ZK6): (**a**) hole position 440 m; (**b**) hole position 430 m; (**c**) hole position 420 m; (**d**) hole position 410 m.

**Figure 16:**
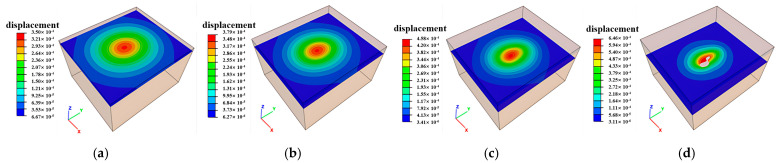
Deformation distribution of surrounding rock during mining of in Block VIII (ZK10): (**a**) hole position 440 m; (**b**) hole position 430 m; (**c**) hole position 420 m; (**d**) hole position 410 m.

Mining-induced stress release and redistribution within surrounding rock masses result in measurable deformations. Horizontal cross-sections extracted at 10 m vertical intervals reveal total deformation distributions in peripheral rock, as illustrated in Figure 14, Figure 15 and Figure 16. Maximum displacements in the ZK4, ZK5, ZK6 (Block II), and ZK10 (Block VIII) goaf regions were observed to not exceed 1 mm, indicating competent rock mass behavior that precludes large-scale instability. However, localized rockfalls and roof collapse may occur in zones with pre-existing discontinuities or stress-induced micro fracturing.

### 3.3. Destruction of Surrounding Rock

**Figure 17:**
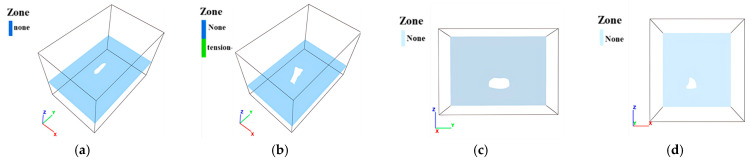
Destruction of surrounding rock of ZK4 mine goaf in Block II: (**a**) hole position 420 m; (**b**) hole position 415 m; (**c**) hole profile x = 562 m; (**d**) hole profile y = 470 m.

**Figure 18:**
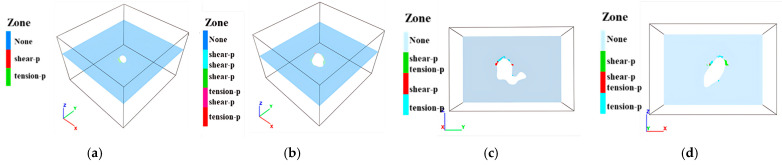
Destruction of surrounding rock in Block II (ZK5 and ZK6): (**a**) hole position 410 m; (**b**) hole position 400 m; (**c**) hole profile x = 430 m; (**d**) hole profile y = 570 m.

**Figure 19:**
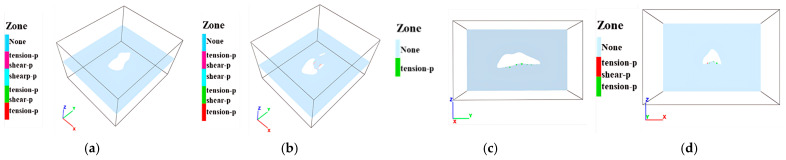
Destruction of surrounding rock in Block VIII (ZK10): (**a**) hole position 440 m; (**b**) hole position 430 m; (**c**) hole position 190 m; (**d**) hole position 450 m.

Horizontal sections of the goaf were analyzed to assess the destruction characteristics of the surrounding rock, as illustrated in Figure 17, Figure 18 and Figure 19. In these figures, blue (None) indicates intact rock, while other colors represent different modes of rock mass failure. In ZK4 (Block II), the surrounding rock exhibits minimal failure, with only sporadic tensile failure elements occurring near the sidewall corners. In contrast, ZK5 and ZK6 (Block II) display widespread tensile and shear failure elements within the sidewall rock at the 410 m and 400 m levels. Vertical section analysis reveals that failure elements predominantly occur in the upper–middle portion of the surrounding rock, with tensile failure elements mainly concentrated in the roof. In ZK10 (Block VIII) at the 390 m level, failure elements are relatively localized, and a limited number of tensile failure elements are distributed in the floor. The numerical results align well with field survey data, indicating reasonable model accuracy. Although no significant ground pressure activity has been observed in the mining area, delayed backfilling in some goafs may contribute to instability. Key influencing factors, including roof exposure area, goaf dimensions, geometry, burial depth, and adjacent goaf interactions, may lead to localized failures in roof and pillar zones. Overall, the current structural integrity of the goafs in the mining area remains relatively unstable.

### 3.4. Surface Subsidence Distribution Characteristics

**Figure 20:**
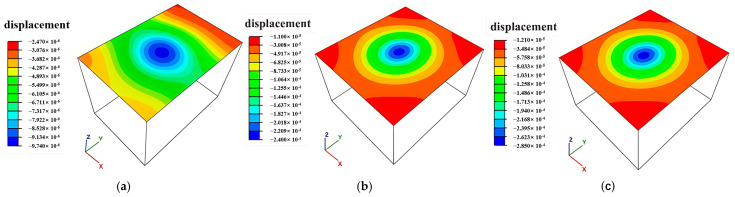
Distribution map of surface subsidence in mine goafs: (**a**) ZK4 in Block II; (**b**) ZK5 and ZK6 in Block II; (**c**) ZK10 in Block VIII.

Prolonged exposure of mine goafs coupled with surface loading exacerbates the development of subsidence over goaf regions, as demonstrated in Figure 20. Progressive extraction of lower ore bodies has formed a subsidence bowl spatially correlated with the planar distribution of goaf clusters. Maximum surface subsidence generally remains minimal, exemplified by 0.28 mm at borehole ZK10 (Block VIII), with negligible deformation observed above the ZK4, ZK5, and ZK6 goafs (Block II), indicating a limited surface impact from current mining operations. At present, the continuum method is adopted in numerical simulation analyses; however, it is difficult to analyze large deformations in rock mass structures. Therefore, the advanced Smoothed Particle Hydrodynamics (SPH) technology [24] should be integrated to research the problems of rock mass landslide and collapse in the goaf regions.

### 3.5. Engineering Validation

InSAR technology has the advantages of high precision and a wide range in surface deformation monitoring, but the results are uncertain due to system errors, environmental interference factors, data processing and algorithm limitations, and differences in deformation interpretation. Numerical simulation can predict surface subsidence and optimize the mining scheme, but there are also model uncertainties, parameter uncertainties, and numerical errors. In order to carry out verification of simulation results in this paper, the engineering site was surveyed.

The historical unregulated cluster mining within the project area has created interconnected goaf regions exceeding 30,000 m^3^ in volume, with vertically overlapping cavities where the minimum roof thickness between adjacent stopes measures approximately 10 m. The absence of original survey data from both surface and underground operations, combined with mining-induced disturbances (mechanical operations and blasting activities) and progressive expansion of lower-level goafs, has triggered stress redistribution. This mechanism induced ground subsidence in shallow historical goafs, compromising the global stability of the mining complex. Surface deformation-induced fissures facilitate groundwater infiltration into rock masses, accelerating weathering processes that degrade the mechanical properties of surrounding rocks, thereby compounding goaf instability. Field investigations corroborated the numerical modeling results, identifying three primary geohazard types: ground subsidence craters, tensile ground fissures, and slope rock mass collapse along mine boundaries. To mitigate these risks, the implementation of high-frequency deformation monitoring coupled with targeted support reinforcement or backfilling measures is imperative for critical goaf zones exhibiting displacement rates exceeding 2 mm/month. Considering the geological and engineering characteristics, the selected index is more stringent than the slope monitoring index (3 mm/month) in the specification [25].

#### 3.5.1. Ground Subsidence and Surface Fissures in Goaf Regions

**Figure 21:**
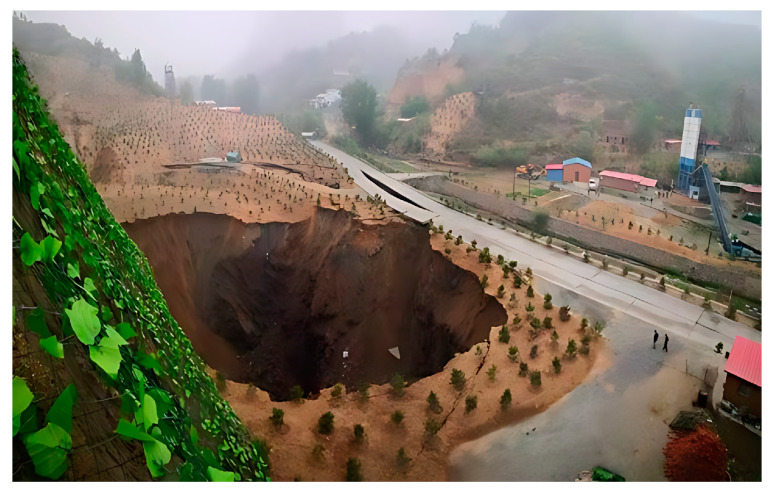
Collapse pit in Block VIII.

Two distinct ground subsidence zones and multiple fissure propagation zones have been identified within the investigation area. The statistical characteristics of surface collapse and subsidence features are documented in Table 4, with representative subsidence and collapse points illustrated in Figure 21.

#### 3.5.2. Slope Collapse in Mining Pit

**Figure 22:**
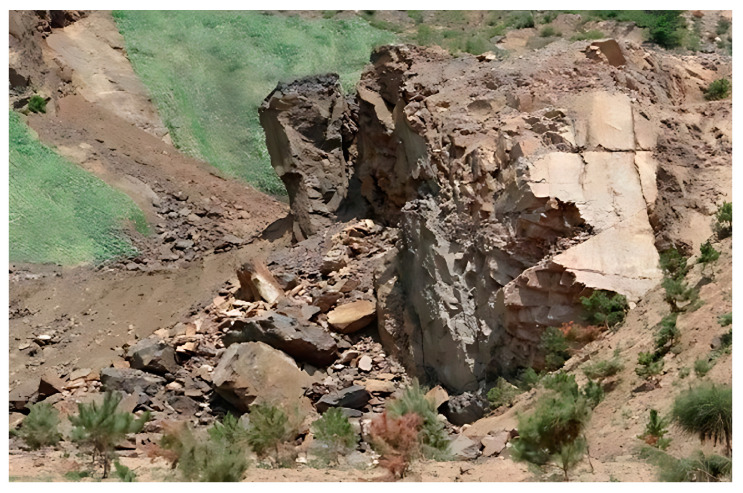
Collapse of mine goaf in Block VIII.

During goaf collapse processes, the surrounding rock masses experience shear failure induced by roof rock punching effects. This results in imbalanced stress distribution around the collapse zones, where tensile stresses develop along the periphery. These stresses exacerbate surface deformation while promoting joint fissure propagation within adjacent rock masses. Unstable rock masses containing outward-dipping structural planes and interconnected fractures are prone to gravitational collapse, with the most severe collapse hazards concentrated in Block VIII, as shown in Figure 22.

## 4. Conclusions

This study investigates the stability of mineral engineering structures in complex mine goaf regions through an integrated approach combining multivariate survey technologies and numerical modeling. The methodology enables the precise identification of goaf orientations and high-fidelity reconstruction of cavity geometries in complex mining environments. FLAC^3D^-based numerical simulations, validated through field investigations, assess structural stability and verify associated geological hazards. Key findings include the following:

(1) A novel integration of surface deformation monitoring techniques—incorporating InSAR, UAV photogrammetry, and terrestrial LiDAR (C-ALS)—successfully identified high-risk goafs, with measured surface deformation rates reaching 14 cm/year. The 3D laser scanning system provides groundbreaking capability for characterizing geometrically complex underground voids, offering new technical solutions for mineral engineering applications.
(2) Stability analysis revealed distinct risk stratification: Goafs in ZK4 (Block II) and ZK10 (Block VIII) exhibit unstable conditions, while those in ZK5 and ZK6 (Block II) of maintain marginal stability. Stress concentrations predominantly occur at geometric discontinuities in goaf sidewalls, where tensile stress development in roof strata precipitates localized rockfall hazards due to the inherently low tensile strength of fractured rock masses.
(3) Field verification confirmed that numerical simulation results accurately predict actual geohazards, including ground subsidence craters and slope collapses that critically compromise mine safety. These findings necessitate implementation of high-frequency deformation monitoring coupled with targeted support measures (reinforcement or backfilling) to mitigate geological risks.

This multi-survey approach demonstrates broad applicability for both metallic and non-metallic mine goaf detection, with potential extensions to soil cave collapse hazard assessment. Notably, while InSAR technology faces limitations in vegetated areas due to coherence loss, the proposed Distributed Scatterer adaptive algorithm robustly estimates covariance matrices, overcoming traditional InSAR constraints in low-vegetation coverage areas. This advancement significantly improves deformation measurement accuracy and computational efficiency compared to conventional Permanent Scatterer techniques.

## Figures and Tables

**Table 1:** Risk classification of the warning level.

Warning Level	I (Low Risk)	II (Medium Risk)	III (High Risk)
Surface deformation rate (mm/year)	<10	10–20	>20
Risk description	The surface deformation is controllable and belongs to normal fluctuation.	The surface deformation is obvious, and there are potential safety hazards.	Severe surface deformation may lead to pavement collapse.
Counter measure	Routine monitoring	Strengthen monitoring frequency	Start emergency reinforcement

**Table 2:** Spatial form parameters of mine goafs.

Main Parameters	Length (m)			Spatial Volume (m^3^)	Accurate Model Shape
	X-Axis	Y-Axis	Z-Axis		
ZK4 in Block II	14.59	21.07	7.35	442.90	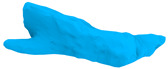
ZK5, ZK6 in Block II	43.35	69.09	49.09	29,280.60	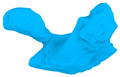
ZK10 in Block VIII	36.60	68.97	27.95	20,097.60	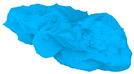

**Table 3:** Geotechnical mechanical parameters.

Name	Modulus of Elasticity (GPa)	Cohesion (MPa)	Internal Friction Angle (°)	Poisson’s Ratio	Compressive Strength (MPa)	Tensile Strength (MPa)	Density (kg/m^3^)
Upper surrounding rock	19.00	2.50	55	0.30	62.50	3.75	2700
Lower surrounding rock	25.43	3.50	65	0.23	96.08	3.34	2900
Magnetite	29.27	4.20	70	0.27	227.00	3.38	3270

**Table 4:** Statistical characteristics of ground collapse and subsidence.

Location	Subsidence Area (m^2^)	Damage Characteristics
Block II	200	Surface subsidence with collapse crater formation, accompanied by roadway degradation
Block VIII	2879	Extensive ground collapse, surface fissure propagation, and localized slope failures

## Data Availability

The data presented in this study are available on request from the corresponding author. The data are not publicly available due to the confidentiality requirements for unfinished research projects.
